# Multidrug-resistant *Neisseria gonorrhoeae* infection with ceftriaxone resistance and intermediate resistance to azithromycin, Denmark, 2017

**DOI:** 10.2807/1560-7917.ES.2017.22.42.17-00659

**Published:** 2017-10-19

**Authors:** David Terkelsen, Jacob Tolstrup, Camilla Hundahl Johnsen, Ole Lund, Helle Kiellberg Larsen, Peder Worning, Magnus Unemo, Henrik Westh

**Affiliations:** 1 Department of Clinical Microbiology, Hvidovre Hospital, Copenhagen, Denmark; 2 These authors contributed equally to the work; 3 Department of Dermatovenerology, Bispebjerg Hospital, Copenhagen, Denmark; 4 Department of Bio and Health Informatics, Technical University of Denmark, Lyngby, Denmark; 5 WHO Collaborating Centre for Gonorrhoea and other Sexually Transmitted Infections, Department of Laboratory Medicine, Faculty of Medicine and Health, Örebro University, Örebro, Sweden; 6 Institute of Clinical Medicine, Faculty of Health and Medical Sciences, University of Copenhagen, Copenhagen, Denmark; 7 These authors share last authorship

**Keywords:** Neisseria gonorrhoeae, STD, multidrug-resistant, antibiotic, Resfinder, treatment

## Abstract

We describe a multidrug-resistant *Neisseria gonorrhoeae* infection with ceftriaxone resistance and azithromycin intermediate resistance in a heterosexual man in Denmark, 2017. Whole genome sequencing of the strain GK124 identified MSLT ST1903, NG-MAST ST1614 and all relevant resistance determinants including similar penA resistance mutations previously described in ceftriaxone-resistant gonococcal strains. Although treatment with ceftriaxone 0.5 g plus azithromycin 2 g was successful, increased awareness of spread of gonococcal strains threatening the recommended dual therapy is crucial.

In the last decade, high-level resistance to extended-spectrum cephalosporins (ESCs) has been reported in *Neisseria gonorrhoeae* worldwide, leading to treatment failures with oral cefixime and the more potent injectable ceftriaxone [1-10]. The emergence of ESC resistance has caused public health concern worldwide [9,11-13] and dual antimicrobial therapy is now recommended as the first-line empirical treatment of uncomplicated gonorrhoea in parts of the world including Europe [8,14]. It is worrying that failure to treat pharyngeal gonorrhoea with dual antimicrobial therapy was recently verified in the United Kingdom [15]. Fortunately, ceftriaxone resistance remains rare in Europe [16].

This report describes one rare case of a multidrug-resistant (MDR) *N. gonorrhoeae* infection with ceftriaxone resistance and intermediate resistance to azithromycin, detected in Denmark in 2017.

## Case description

A heterosexual man in his 20s from Denmark presented at a general practitioner clinic in Copenhagen, Denmark in January, 2017 with urethritis symptoms that he had had for two days. He had had three unprotected sexual relations with women (of Danish, Chinese and Australian nationality) during the previous 6 months and he had not travelled abroad during that time. He was empirically treated unsuccessfully with a single oral dose of azithromycin 1 g because non-gonococcal urethritis was suspected. A nucleic acid amplification test (NAAT) (Aptima Combo 2, Hologic Inc.) of urine sampled at the visit to the general practitioner clinic, was positive for *N. gonorrhoeae* and the patient was referred 6 days later to the sexually transmitted diseases (STD) clinic with urethritis symptoms without purulent discharge. Culture from a urethral swab (eight days after symptom onset) was positive for *N. gonorrhoeae*, while NAAT and culture of pharyngeal and rectal samples were negative. He was successfully treated with single doses of ceftriaxone 0.5 g intramuscularly and azithromycin 2 g orally and advised to abstain from sexual contacts until test-of-cure (TOC) as outlined in the Danish treatment recommendation [17]. Two weeks after treatment, the TOC using NAAT of urethral and pharyngeal samples were negative and all symptoms were resolved. The patient had no sexual contacts between onset of symptoms and TOC. The Danish sexual contact was NAAT-negative and the two other women were lost to follow-up.

## Characterisation of the *Neisseria gonorrhoeae* isolate

Minimum inhibitory concentration (MIC) values for ceftriaxone, cefixime, azithromycin, spectinomycin, ciprofloxacin, and benzylpenicillin in the *N. gonorrhoeae* strain isolated from the patient, GK124, were determined in duplicate using Etest (bioMerieux, Ballerup, Denmark) and results were interpreted in accordance with the European Committee on Antimicrobial Susceptibility Testing (EUCAST) interpretative criteria [18]. GK124 showed resistance to ceftriaxone (MIC: 0.25–0.5 mg/L), cefixime (MIC: 1 mg/L), ciprofloxacin (MIC: > 32 mg/L) and benzylpenicillin (MIC: > 256 mg/L), and intermediate resistance to azithromycin (MIC: 0.5 mg/L). GK124 was susceptible to spectinomycin (MIC: 8 mg/L) (Table).

**TABLE:** Antibiotic resistance pattern, multidrug-resistant *Neisseria gonorrhoeae* patient isolate GK124*,* Denmark, 2017, compared with four whole-genome-sequenced ceftriaxone-resistant *N. gonorrhoeae* strains

Strain (year, country, name)	2017, Denmark, GK124	2015, Japan, FC428 [7]	2013, Australia, A8806 (WHO Z [2,20])	2010, France, F89 (WHO Y [1,20])	2009, Japan, H041 (WHO X [5,20])
Minimal inhibitory concentration [mg/L^a^]					
Ceftriaxone	0.25–0.5	0.5	0.5	1–2	2–4
Cefixime	1	1	ND	4	8
Azithromycin	0.5	0.25	0.25	1	1
Spectinomycin	8	8	≤ 64	16	16
Ciprofloxacin	> 32	> 32	>32	> 32	> 32
Benzylpenicillin	> 256	> 32	1	1	4
Sequence type					
MLST	1903	1903	7363	1901	7363
NG-MAST	1614	3435	4015	1407	4220
Key ceftriaxone resistance alterations in PBP2					
Encoded by *penA*	A311V, T483S (I312M, V316T, G545S)^b^	A311V, T483S (I312M, V316T, G545S)^b^	A311V, T483S (I312M, V316T, G545S)^b^	A501P (I312M, V316T, G545S)^b^	A311V, V316P, T483S (I312M, G545S)^b^
Other relevant resistance determinants					
PorB1b	G120K, A121D	G120K, A121D	G120K, A121D	G120K, A121N	G120K, A121D
*mtr* locus	A-del	A-del	ND	A-del	A-del
GyrA	S91F, D95A	ND	S91F, D95N	S91F, D95G	S91F, D95N

MIC: minimum inhibitory concentration; MLST: multilocus sequence typing; ND: not described; NG-MAST: *N. gonorrhoeae* multi-antigen sequence typing; PBP2: penicillin-binding protein 2; WHO: World Health Organization reference strain.

^a^ Only whole MIC doubling dilutions are reported.

^b^ Mutations in the parenthesis indicate a mosaic *penA* allele.

Whole genome sequencing (WGS) was performed on an Illumina MiSeq as previously described [19]. The GK124 genome was compared with previously described ceftriaxone-resistant strains (WHO X (H041), WHO Y (F89), WHO Z (A8806) [1,2,5,20] and FC428 [7]) using ResFinder 3.0, an update of ResFinder focusing on chromosomal mutational resistance (cge.cbs.dtu.dk/services/ResFinder/version 3.0) and previous publications (Table). Based on the WGS sequence, the sequence type (ST) of GK124 was ST1903 as derived by multilocus sequence typing (MLST) and ST1614 as derived by *N. gonorrhoeae* multi-antigen sequence typing (NG-MAST) (Table). The draft genome of GK124 can be found under GenBank accession number: PRJEB22246.

Regarding ESC resistance determinants, GK124 contained the amino acid alterations A311V, I312M, V316T, T483S and G545S, which indicated that the strain had a mosaic *penA* allele as well as additional key ceftriaxone resistance mutations [4,5,7,13,20]. Furthermore, the strain contained G120K and A121D alterations in PorB1b and the A-deletion in the inverted repeat sequence of the *mtrR* promoter region that further increase the ESC MICs and contribute to the MDR characteristics of GK124. No mutations associated with azithromycin resistance were found in the 23S rRNA gene and, accordingly, the intermediate resistance to azithromycin was due to the *mtrR* resistance determinant and possibly additional unknown mutations. SNPs in *gyrA* (S91F, D95A), encoding the A subunit of the DNA gyrase, caused the high-level ciprofloxacin resistance [4] (Table).

## Discussion

This report describes one rare case of MDR *N. gonorrhoeae* infection with ceftriaxone resistance and intermediate resistance to azithromycin in Denmark, 2017. This case was reported by the national healthcare authorities but has not lead to wider public health actions [21]. Increased awareness of the spread of this type of gonococcal strains that threaten the recommended dual antimicrobial therapy is crucial. This is especially important when the incidence of gonorrhoea is rapidly increasing in many countries, including in Denmark where a sevenfold increase in the gonorrhoea incidence was observed from 2011 to 2016 [21]. This increase in Denmark was not caused by increased awareness as the number of samples tested per year has increased by about 2% and dual testing for chlamydia and *N. gonorrhoeae* by NAAT is implemented in most of the country. Furthermore, dual antimicrobial therapy with ceftriaxone 500 mg plus azithromycin 2 g for empiric treatment of gonorrhoea is recommended in Europe [14]; however, the level of implementation of this dual therapy is unknown and several countries still recommend monotherapy with ceftriaxone [22] and even other antimicrobials such as azithromycin.

The MLST ST of GK124, ST1903, has previously been described in the ceftriaxone-resistant strain FC428 isolated in 2015 in Japan [7], but the NG-MAST ST1614 has not been previously associated with ceftriaxone resistance. GK124 contained several key ESC resistance mutations in *penA*: I312M, V316T, G545S, which are indicator mutations for a mosaic *penA* allele, and also two (A311V, T483S) of the three key ceftriaxone resistance mutations in the pan-resistant H041 (WHO X) [5,13,20]. These mutations have also been found in most of the ceftriaxone-resistant strains characterised in detail [2,5,7,20]. This provides further evidence that both ceftriaxone-resistant strains and ceftriaxone resistance-determining PBP2 sequences might be spreading internationally [7].

## Conclusion

An MDR *N. gonorrhoeae* strain with ceftriaxone resistance and intermediate resistance to azithromycin was found in Denmark in 2017. Our patient only had genital infection and was cured by dual antimicrobial therapy. Increased awareness of the spread of this type of gonococcal strains, improved implementation of the recommended dual antimicrobial therapy (for all patients and not only for high-risk populations), partner notification, TOC and appropriate verification/falsification of suspected treatment failures are crucial internationally as the recommended dual antimicrobial therapy is threatened. Ultimately, novel treatment options for gonorrhoea and ideally an effective gonococcal vaccine are imperative.
